# Tic Douloureux, or Neuralgia Facialis, and Other Nervous Affections; Their Seat, Nature, and Cause; with Cases Illustrating Successful Methods of Treatment

**Published:** 1841-10

**Authors:** 


Art. XVI.—*Tic Douloureux, or Neuralgia Facialis, and other Nervous Affections; their Seat, Nature, and Cause; with Cases illustrating successful Methods of Treatment.* By A. H. Allnatt, m.d. a.m.—*London,* 1841. 8vo, pp. 184.

We are much at a loss to know what can have induced Dr. Allnatt to publish the present volume. He must be well aware that it contains not a particle of novelty in doctrine or practice; and since the appearance of Dr. Rowland's very excellent Treatise on Neuralgia generally, there is no want of a compendium of our actual knowledge of the subject, as far as this is possessed by Dr. Allnatt. Had he, indeed, been acquainted with M. Valleix's recent most elaborate *Traitê des Nevralgies,* and given some interest to his barren pages by transferring to them some of the new facts and deductions to be found therein, he might have advanced a better claim to the notice of the profession. As he has not done this, we shall ourselves give a full analysis of M. Valleix's treatise in our next Number; and in the meantime would recommend Dr. Allnatt not only to study it, but to study much longer the great book of nature, before he again ventures to commit his reputation with the public in a treatise on neuralgia,

Dr. Allnatt informs us that he is "firmly of opinion that Tic Douloureux arises, *in every instance,* from an unhealthy condition of the digestive apparatus," particularly of the liver, which unhealthy condition is elsewhere stated to be an "irritation," inducing, "in cases of long standing, hyperæmia." This irritation, of course, acts on the cerebro-spinal axis, and gives rise to the neuralgia at the peripheral extremities of the nerves. Dr. Allnatt's treatment, in accordance with this old and well-known theory, is precisely that of Abernethy and Sir Charles Bell, more especially of the latter author, as croton oil is his grand dependence. "I have found," he says, "the free use of aperients of *unfailing efficacy,* and I give a decided preference, over all others, to a pill combining a small quantity of croton oil with stomachic aperients." That this treatment has been successful, in a certain proportion of cases, has been shown by Sir Charles Bell and others, and that it is very proper, in certain circumstances, we do not attempt to deny; but we venture to pronounce, from our own experience in this distressing malady, probably somewhat longer than Dr. Allnatt's, that ere many years have passed over his head, he will be less ready to speak of its "unfailing efficacy." Dr. Allnatt's book might have been acknowledged to be a respectable thesis at graduation, but he has assuredly been ill-advised in claiming public attention to it.

